# Enhancing poultry health management through machine learning-based analysis of vocalization signals dataset

**DOI:** 10.1016/j.dib.2023.109528

**Published:** 2023-08-28

**Authors:** Segun Adebayo, Halleluyah O. Aworinde, Akinwale O. Akinwunmi, Olufemi M. Alabi, Adebamiji Ayandiji, Aderonke B. Sakpere, Adetoye Adeyemo, Abel K. Oyebamiji, Oke Olaide, Echentama Kizito

**Affiliations:** a College of Computing and Communication Studies, Bowen University, Iwo Nigeria; b College of Agriculture, Engineering and Science, Bowen University, Iwo Nigeria; c Computer Science Department, University of Ibadan, Ibadan Nigeria

**Keywords:** Audio, Dataset, Signals, Machine learning, Disease

## Abstract

Population expansion and rising consumer demand for nutrient-dense meals have both contributed to an increase in the consumption of animal protein worldwide. A significant portion of the meat and eggs used for human consumption come from the poultry industry. Early diagnosis and warning of infectious illnesses in poultry are crucial for enhancing animal welfare and minimizing losses in the breeding and production systems for poultry. On the other hand, insufficient techniques for early diagnosis as well as infectious disease control in poultry farms occasionally fail to stop declining productivity and even widespread death.

Individual physiological, physical, and behavioral symptoms in poultry, such as fever-induced increases in body temperature, abnormal vocalization due to respiratory conditions, and abnormal behavior due to pathogenic infections, frequently represent the health status of the animal. When birds have respiratory problems, they make strange noises like coughing and snoring. The work is geared towards compiling a dataset of chickens that were both healthy and unhealthy.

100 day-old poultry birds were purchased and split into two groups at the experimental site, the poultry research farm at Bowen University. For respiratory illnesses, the first group received treatment, whereas the second group did not. After that, the birds were separated and caged in a monitored environment. To eliminate extraneous sounds and background noise that might affect the analysis, microphones were set a reasonable distance away from the birds. The data was gathered using 24-bit samples at 96 kHz. For 65 days, three times per day (morning, afternoon, and night) of audio data were continually collected. Food and water are constantly provided to the birds during this time. During this time, the birds have constant access to food and water. After 30 days, the untreated group started to sound sick with respiratory issues. This information was also noted as being unhealthy. Chickens' audio signals were recorded, saved in MA4, and afterwards converted to WAV format.

This dataset's creation is intended to aid in the design of smart technologies capable of early detection and monitoring of the status of birds in poultry farms in a continuous, noninvasive, and automated way.

**Specification Table**

**Table:** 

Subject	Applied Machine Learning
Specific Subject Area	Signal Processing
Type of Data	Audio Signal
How data were acquired	100 day-old poultry birds were purchased and split into two groups at the experimental site, the poultry research farm at Bowen University. For respiratory illnesses, the first group received treatment, whereas the second group did not. After that, the birds were separated and caged in a monitored environment. To eliminate extraneous sounds and background noise that might affect the analysis, microphones were set a reasonable distance away from the birds. The data was gathered using 24-bit samples at 96 kHz. For 65 days, three times per day (morning, afternoon, and night) of audio data were continually collected. Food and water are constantly provided to the birds during this time. During this time, the birds have constant access to food and water. After 30 days, the untreated group started to sound sick with respiratory issues. This information was also noted as being unhealthy. Chickens' audio signals were recorded, saved in MA4, and afterwards converted to WAV format.
Data format	Raw Audio Signal
Description of Data Collection	There are 346 audio signal files altogether in the dataset, and they are organized into three folders: healthy, noisy, and sick. There are 139 audio files in the healthy folder, 86 in the noise folder, and 121 in the unhealthy folder. Wav files are used to store the audio files.
Data source Location	• Institution: Bowen University, Poultry Research Farm, Bowen University Commercial Farm • City/Town/Region: Iwo • Country: Nigeria
Data Accessibility	Repository name: Mendeley Data Data identification number: doi: Direct URL to data: doi:10.17632/zp4nf2dxbh.2, https://dx.doi.org/10.17632/zp4nf2dxbh.1

## 1 Value of the Data


 

• Audio signal dataset of the chicken fecal have potential value in early detection mechanisms and diagnosis of poultry diseases due to high contagious rate of such diseases.
• Respiratory related diseases have been discovered to have damaging effects on the vocal tract of poultry and which thereby affect both their breathing behaviour and the sound they produced. Hence, early observation and classification for vocal data gotten from them is imperative.
• There is paucity of publicly available datasets of poultry vocalization signals for respiratory related diseases in poultry. Making annotated dataset gotten from this work available will promote feasibility, accessibility, interoperability, and reproducibility (FAIR Data) a possibility.
• The dataset is applicable in the development of computer vision-based models for audio signals, segmentation, and classification.
• The dataset will give more insights beyond human physical observation for accurate and precise detection of poultry diseases. It will aid agricultural extension agents in engaging poultry farmers in a more effective training and orientation.

## 2 Objective

Due to issues with stocking density and inadequate management brought on by the growth of the large-scale and intensive broiler business, the incidence of respiratory illnesses such as infectious bronchitis, Newcastle disease, and avian influenza has grown. When broilers have respiratory conditions, they will make strange noises like coughing and snoring.

Animals' vocalizations offer a wealth of information about their well-being, emotions, and behavior [1]. These have propelled many researchers to use various techniques for analyzing animal vocalizations in recent years [2].

Acoustic technology has been used to analyze growth rate in poultry birds [3], [4], disease detection [5], [6], [7], sex of birds [8], poultry birds under heat or fear stress [9], [10], [11]. Despite the fact that these studies have demonstrated the value of using animal sound analysis as a tool for early disease, stress, and behavior detection in several animal species, unfortunately, many of these datasets are not publicly available on open source repositories [12]. This study aims at addressing this gap by providing a labeled auditory dataset of healthy and unhealthy chicken sounds for commonly reared poultry birds’ species in Africa on an open-source platform.

## 3 Data Description

The dataset contains 346 audio signal files and grouped into three folders: healthy, noisy, and unhealthy. There are 139 audio files in the healthy folder, 86 in the noise folder, and 121 in the unhealthy folder. The audio signal file has a range of 5 s to 60 s length. The selected sound segments in the unhealthy folder include chicken cough, snore and rale sound. The Noise folder contain sound segments that include background noises (e.g., moving vehicle and human voices) and sound created by poultry bird activities (e.g., feeding, pecking one another). The files are stored in .wav audio format.

In order to investigate, the audio signal was analyzed in the time and frequency domains. The power spectra of the two signals (healthy and unhealthy) were compared in the frequency domain. While the normal sound has more frequency content exactly around the y-axis, the anomalous sound exhibits a noticeable spike of about 0.2 radians.

## 4 Experimental Design, Materials and Methods

### 4.1 Field Data Collection

**Fig. 1:**
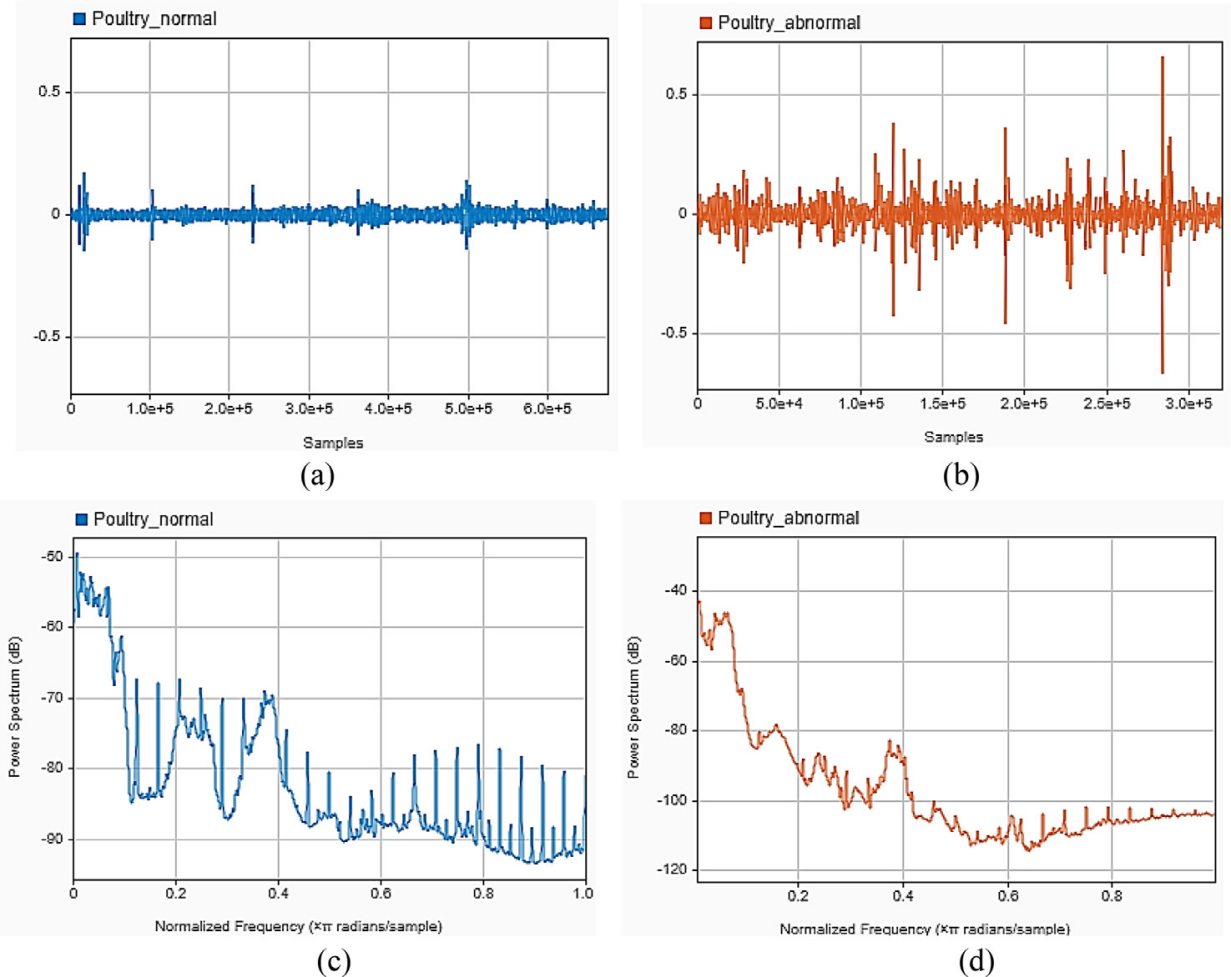
Poultry Sound in time and frequency domain (a) Normal Poultry in time domain, (b) Abnormal Poultry Sound in time domain

**Fig. 2:**
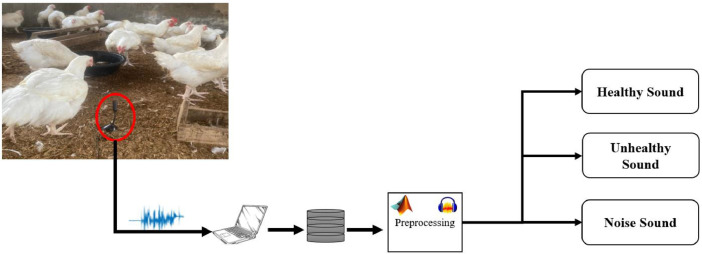
Data acquisition experimental setup.

100 day-old poultry birds were purchased and split into two groups at the experimental site, the poultry research farm at Bowen University. For respiratory illnesses, the first group received treatment, whereas the second group did not. After that, the birds were separated and caged in a monitored environment. To eliminate extraneous sounds and background noise that might affect the analysis, microphones were set a reasonable distance away from the birds. The data was gathered using 24-bit samples at 96 kHz. For 65 days, three times per day (morning, afternoon, and night) of audio data were continually collected. Food and water are constantly provided to the birds during this time. During this time, the birds have access to food and water constantly. After 30 days, the untreated group started to sound sick with respiratory issues. This information was also noted as being unhealthy. Chickens' audio signals were recorded, saved in MA4, and afterwards converted to WAV format (Figs. 1,2).

### 4.2 Data Preprocessing

The sound samples were framed and filtered as part of the sound signal preprocessing. Longer period nonstationary sound samples were then framed by a shifting Hamming window to create a 10 to 30 s stationary signal.

The Hann window [13] is expanded by the Hamming window in that it is a higher cosine window of the same type. The Hamming window is given as:

(1) $$h\left(n\right)=\alpha +\left(1.0-\alpha \right)\cos\left[\left(\frac{2\pi }{N}\right)\right]$$

with a matching form spectrum

(2) $$H\left(\theta \right)=\alpha D\left(\omega \right)+\frac{\left(1.0-\alpha \right)}{2}\left[D\left(\omega +\frac{2\pi }{N}\right)\right]$$

αenables the destructive sidelobe cancellation to be optimized. The window is known as the Hamming window and has the form when the value of is roughly 0.54.

(3) $$H\left(\theta \right)=0.54+0.46cos\left[\left(\frac{2\pi }{N}\right)n\right],\;0\leq n\leq N$$

The window's size is L=N+1.

In order to improve the signal-to-noise ratios of the acoustic samples, filtering was done using the Kalman filter. This is a nonstationary, recursive filter that enables an estimate of the usable signal in noisy time series at each instant of time [14]. Two linear difference stochastic equations define the Kalman technique for digital filtering signal $\left\{x_{k}\right\}$ in steady state.

$$x_{k}=Fx_{k-1}+Gw_{k-1}$$

(4) $$y_{k}=\;Hx_{k}+v_{k}$$

The first equation represents the signal generation process, whereas the second equation describes the signal measurement process.

The measurement noises are represented by the random variables$\;w_{k}$ and $v_{k}$, respectively. These variables can be assumed to be white, unrelated to one another, and to have normal probability distributions.

In the absence of a driving function or process noise, the matrix F links the state at time step 'k-1′ to the state at step 'k'. Also, the matrix H relates the state $x_{k}\;$to the measurement $y_{k}$*.*

At step 'k', the Kalman filter makes an estimate of the process state and subsequently receives input in the form of (noisy) measurements.

Thus, two groups of Kalman filter equations can be distinguished, as shown in equations 5 and 6.

Equation 5 is the time update, while equation 6 is the measurement update:

$${\hat{x}}_{k|k-1}=F{\hat{x}}_{k-1|k-1}$$

(5) $$P_{k|k-1}=FP_{k-1|k-1}F^{T}+Q$$

$$\varkappa_{k}=P_{k|k-1}H_{k}^{T}{\left[H_{k}P_{k|k-1}H_{k}^{T}+R\right]}^{-1}$$

$${\hat{x}}_{k|k}={\hat{x}}_{k|k-1}+\varkappa_{k}\left(y_{k}-H_{k}{\hat{x}}_{k|k-1}\right)$$

(6) $$P_{k|k}=\left[l-\varkappa_{k}H_{k}\right]P_{k|k-1}$$

Where matrix $P_{k|k-1}$ is a priori estimate of error covariance.

## Ethics Statements

The Bowen University Research Ethical Board examined and approved the animal study. The research was conducted under the required ethical guidelines issued and signed by the Director of Research and Strategic Partnership for the Board.

## Credit Author Statement

**Halleluyah Aworinde, Segun Adebayo, Olufemi Alabi, Adebamiji Ayandiji, Echentama Kizito, Oke Olaide and Adetoye Adeyemo** participated in data collection and labelling of the image dataset collected from Bowen University Research and Commercial Poultry Farms.

**Segun Adebayo, Halleluyah Aworinde, Akinwale Akinwunmi, Aderonke Sakpere and Abel Oyebamiji** segmented and annotated the audio signals, and took lead in writing and proof reading the manuscript.

All of the authors contributed to the manuscript and gave their approval to the final version after offering constructive criticism and helping to develop the research, analysis, and manuscript.

## Data Availability

Poultry Vocalization Signal Dataset for Early Disease Detection (Original data) (Mendeley Data).
